# Development and validation of derivative UV spectroscopic method for simultaneous estimation of nicotinamide and tretinoin in their binary mixtures and pharmaceutical preparations

**DOI:** 10.1186/s13065-022-00809-x

**Published:** 2022-03-18

**Authors:** Nazira Sarkis, Abdulkader Sawan

**Affiliations:** Department of Analytical and Food Chemistry, Faculty of Pharmacy, University of Aleppo, Aleppo, Syria

**Keywords:** UV spectroscopy, Tretinoin, Nicotinamide, First-order derivative, Second-order derivative, Synthetic mixture, Pharmaceutical preparations

## Abstract

An accurate, precise, sensitive, and simple spectroscopic method was developed and validated for simultaneous quantification analysis of tretinoin (TRT) and nicotinamide (NCT) with a ratio of 1:40 (TRT: NCT) in a synthetic mixture from dermal pharmaceutical preparations (solution and cream). Wavelengths were chosen in the first and second-order derivatives which are valid for the determination of NCT with the existence of TRT and excipients of the tested pharmaceutical preparations. Wavelength 253 nm was picked for the first-order derivative. Wavelengths 245 and 269 nm were picked for the second derivative. All previous wavelengths were zero-crossing points for TRT and its pharmaceutical preparations. Zero-order spectroscopy was used to determine TRT at the wavelength 348 nm, where no interference with NCT or any substance in the previous pharmaceutical preparation. The linearity range was studied and found to be 20–120 μg/mL and 0.5–5.0 μg/mL for NCT and TRT respectively. The correlation coefficient was 0.9995–0.9999 for NCT and 0.9998–0.9999 for TRT. The limit of detection (LOD) and the limit of quantification (LOQ) of NCT were 1.510 μg/mL and 4.590 μg/mL respectively at the wavelength 269 nm of the second-order derivative.

## Introduction

**Fig. 1:**
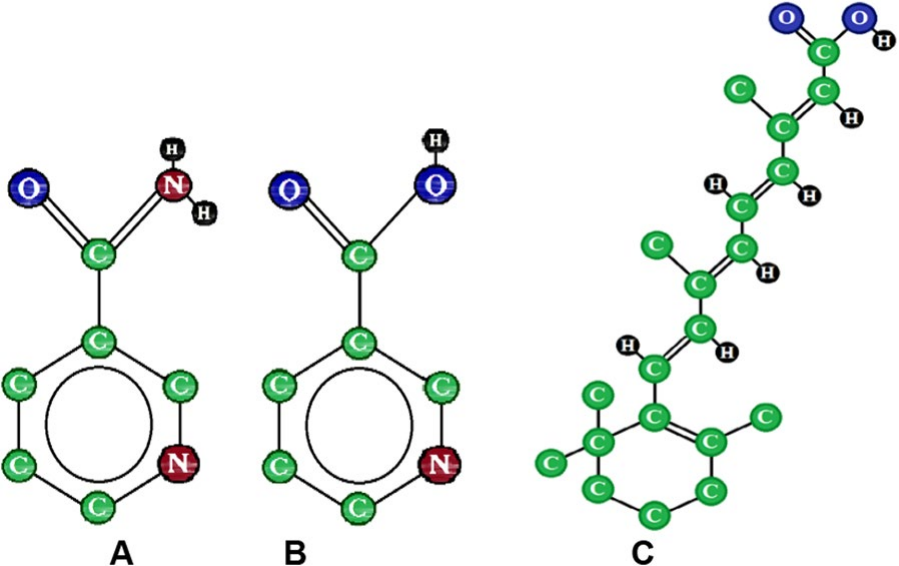
The chemical structures of nicotinamide (**A**), nicotinic acid (**B**), and tretinoin (**C**)

Nicotinamide (NCT), also called niacinamide is 3-pyridine carboxamide Fig. 1-A [1]. NCT is an amide form of nicotinic acid or niacin Fig. 1-B. NCT is white crystalline powder or colorless crystals. it has a molecular weight of 122.12 g/mol. It is also one of the hydrophilic B vitamins, called vitamin B_3_ [2].

Many multivitamins and supplementary pharmaceutical preparations contain NCT. It is also found in many dermal preparations, such as solutions, creams, and gels whether as a single active ingredient or with other active ingredients. NCT has concentrations of 3, 4, or 5% (w/w%) in the previous preparations, which are used for many skin conditions including acne [3].

Tretinoin (TRT) is an all-trans-retinoic acid, as shown in Fig. 1-C [4]. It is a first-generation carboxylic form of retinoid, which is a derivative of vitamin A. TRT has a molecular weight of 300.4 g/mol. It is a yellow powder. Furthermore, it is sensitive to light, heat, and oxygen in the air, especially in solutions. TRT is used for several skin conditions, such as acne (as a first-line treatment), psoriasis, and photoaging. It exists as a single active ingredient or with other active ingredients. Its concentration in solution, cream, and gel preparations is 0.025, 0.050, or 0.100% [5–7].

TRT can be found in combination with clindamycin or benzoyl peroxide [7]. However, there is no marketed international combination of NCT and TRT spread worldwide. Such a combination may only be found locally, like in the United States, for example, it is found as a compounded drug, which is marketed and distributed by Sincerus® Florida, LLC [8]. According to a study, a combination containing NCT and retinol (an alcoholic form of vitamin A) has good therapeutical potentials for acne [9]. Therefore, we developed an ultra-violet (UV) spectroscopy method to estimate NCT and TRT simultaneously in synthetic mixtures and pharmaceutical preparations.

There are many UV spectroscopic methods [10–13] in addition to high-performance liquid chromatography (HPLC) [14–19] and electrochemical methods [20–22], to determine NCT or TRT in combination with other active ingredients. However, no previous studies have developed a method to estimate NCT and TRT simultaneously in a binary mixture or in a pharmaceutical preparation.

The first and second-order derivative methods are simple and accurate for the direct determination of more than one ingredient in mixtures and pharmaceutical preparations, such as TRT with clindamycin [23].

NCT is freely soluble in water, ethanol, and methanol, while slightly soluble in diethyl ether. On the other hand, the solubility of TRT is limited as it is only soluble in dimethyl sulfoxide; slightly soluble in polyethylene glycol 400, octanol, and ethanol; and practically insoluble in mineral oil, glycerin, and water. It is also slightly soluble in methanol, which is used in many developed methods in previous studies since it overcomes ethanol in terms of TRT solubility [11, 24–26]. Thus, methanol is used as a solvent in this suggested method.

The aqueous and alcoholic solutions of NCT, such as methanol, are transparent. NCT does not absorb visual light. Instead, it has a sharp absorbance peak in the UV domain at 262 nm [12, 27]. TRT absorbs visual light with wavelengths less than 440 nm. It has a wide peak around 348 nm (basically between 340 and 353 nm) [11, 23, 28]. According to the previous studies, the spectrum of TRT and its minor absorbance in the rest of the UV domain overlaps NCT. In contrast, NCT has no absorbance at wavelengths higher than 300 nm; thus, NCT does not interfere with the signal of TRT.

## Materials and methods

### Instruments

The ultra-violet spectrophotometric instrument is T80 + UV/V Spectrophotometer Instrument Ltd (UK). It is connected to a computer. The cells used are 1-cm width quartz cells. The weighing device is an analytical balance (Sartorius, model 2474, Germany). Other devices and instruments used to achieve the work are an ultrasonic bath (Power sonic, model 405, Korea), a centrifuge device (90-1 Centrifuge, Shanghai Surgical Instruments Factory, China), a porcelain mortar, volumetric dark flasks, and several scales of glass pipettes.

### Solvents and chemicals

Standard active pharmaceutical ingredients are nicotinamide powder 99% (BDH Laboratory Supplies, England), tretinoin powder ≥ 98% gifted by Rama Pharma Co, Aleppo, and methanol of analytical grade (Merck, Germany).

### Preparation of standard solutions

#### Standard solution of nicotinamide

First, 20 mg of NCT was weighed. Then it was transferred into a 20-mL flask and diluted with methanol to the mark to obtain a standard stock solution of NCT with a concentration of 1000 µg/mL. Then, six quantities of 0.2, 0.4, 0.6, 0.8, 1.0, and 1.2 mL were pipetted out to 10-mL flasks and diluted with methanol to prepare a series of standard solutions of NCT with concentrations of 20, 40, 60, 80, 100, and 120 µg/mL.

#### Standard solution of tretinoin

After weighing 20 mg of TRT, it was transferred into a 20-mL flask and diluted with methanol to obtain a standard stock solution of TRT with a concentration of 1000 µg/mL. Then 0.5 mL was pipetted out to a 20-mL flask to obtain a stock solution of TRT with a concentration of 25 µg/mL. Then, ten quantities from 0.2 to 2.0 mL were pipetted out to 10-mL flasks and diluted with methanol to prepare stock solutions of TRT with concentrations of 0.5, 1.0, 1.5, 2.0, 2.5, 3.0, 3.5, 4.0, 4.5, and 5.0 µg/mL.

#### Preparation of standard mixture of nicotinamide and tretinoin

The synthetic mixture of NCT and TRT was made in the ratio of 1:40 (TRT: NCT). The same procedure was followed in Sects. 2.3.1 and 2.3.2. Except that the same final 20-mL flask was used for both NCT and TRT with concentrations of 1000 µg/mL and 25 µg/mL respectively.

### Preparation of synthetic mixture from pharmaceutical formulas

#### Solution formula

Locacid®, a dermal solution formula which is produced by Universal Pharma Co, Damascus. It is labeled to contains 1000 µg/mL of TRT (0.1% w/v%). The volume of the container is 30 mL. Two quantities of 0.5 mL of the dermal solution were pipetted. Each one was transferred to a 20-mL flask. A quantity of 20 mg of NCT was weighed and transferred to one of the previous flasks. Both flasks were diluted with methanol to obtain two work solutions. The first one (A) contains a synthetic mixture of; the dermal solution formula, which includes excipients and TRT, plus NCT with a ratio of 1:40 (TRT: NCT) (the same ratio as if the formula also contains 4% of NCT). The second one (B) only contains the formula of the dermal solution which includes excipients and TRT. This procedure was done to study the absorbance of all ingredients of the formula and compare it to the absorbance of the standard solution of TRT. Next, two quantities of 0.4 mL were pipetted from A and B. Each of them was transferred to 4 of 10-mL flasks to obtain 8 solutions. Four of them (A series) contain TRT, excipients, and NCT, while the others (B series) contain only TRT and excipients. Standard additions were added before diluting with methanol. Standard additions of 0%, 50%, 100%, and 150% of NCT and TRT were added to the A series to obtain final concentrations of 40, 60, 80, and 100 µg/mL and 1.0, 1.5, 2.0, and 2.5 µg/mL of NCT and TRT respectively. Standard additions of 0%, 50%, 100%, and 150% of TRT were added to the B series to obtain final concentrations of 1.0, 1.5, 2.0, and 2.5 µg/mL of TRT. The flask with a concentration of 1.0 µg/mL of TRT which has no standard addition was given according to the labeled 0.1% concentration of the formula.

#### Cream formula

Retinoram®, a dermal cream formula which is produced by Rama Pharma Co, Aleppo. It is labeled to contain 1000 µg/g of TRT (0.1% w/w%). The net weight of the cream is 30 g. Two quantities of 500 mg of the cream were weighed. Each one was transferred to a mortar with 10 mL of methanol for trituration then transferred to a 20-mL flask. Next, each of them was sonicated for 10 min to ensure the maximum disintegration of the cream and full dissolution of TRT. After sonication, a quantity of 20 mg of NCT was weighed and added to one of the previous flasks. Both were diluted with methanol. Then, each of them was centrifuged to obtain two work solutions. The first one (A) contains a synthetic mixture of; the cream formula, which includes excipients and TRT, plus NCT with a ratio of 1:40 (TRT: NCT) (the same ratio as if the formula also contains 4% of NCT). The second one (B) only contains the formula of the cream which includes excipients and TRT. Next, two quantities of 0.4 mL were pipetted from A and B. Each of them was transferred to 4 of 10-mL flasks to obtain 8 solutions. Four of them (A series) contain TRT, excipients, and NCT, while the others (B series) contain only TRT and excipients. Standard additions were added before diluting with methanol. Standard additions of 0%, 50%, 100%, and 150% of NCT and TRT were added to the A series to obtain final concentrations of 40, 60, 80, and 100 µg/mL and 1.0, 1.5, 2.0, and 2.5 µg/mL of NCT and TRT respectively. Standard additions of 0%, 50%, 100%, and 150% of TRT were added to the B series to obtain final concentrations of 1.0, 1.5, 2.0, and 2.5 µg/mL of TRT. The flask with a concentration of 1.0 µg/mL of TRT which has no standard addition was given according to the labeled 0.1% concentration of the formula.

## Results and discussion

### First and second-order derivative spectrophotometric optimal wavelengths

**Fig. 2:**
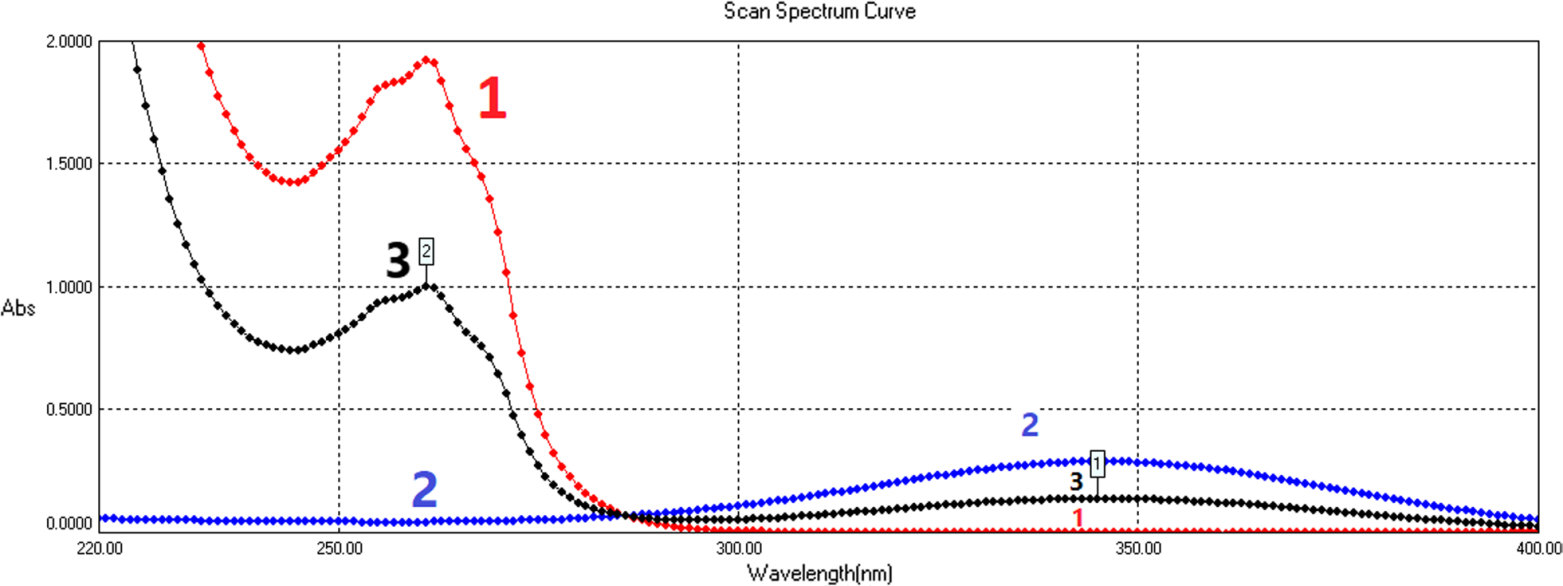
The zero-order UV absorption spectrogram in methanol shows: (1) nicotinamide (80 µg/mL) with 262 nm peak, (2) tretinoin (2 µg/mL) with 348 nm peak, and (3) their binary mixture (NCT 40 µg/mL + TRT 1 µg/mL)

**Fig. 3:**
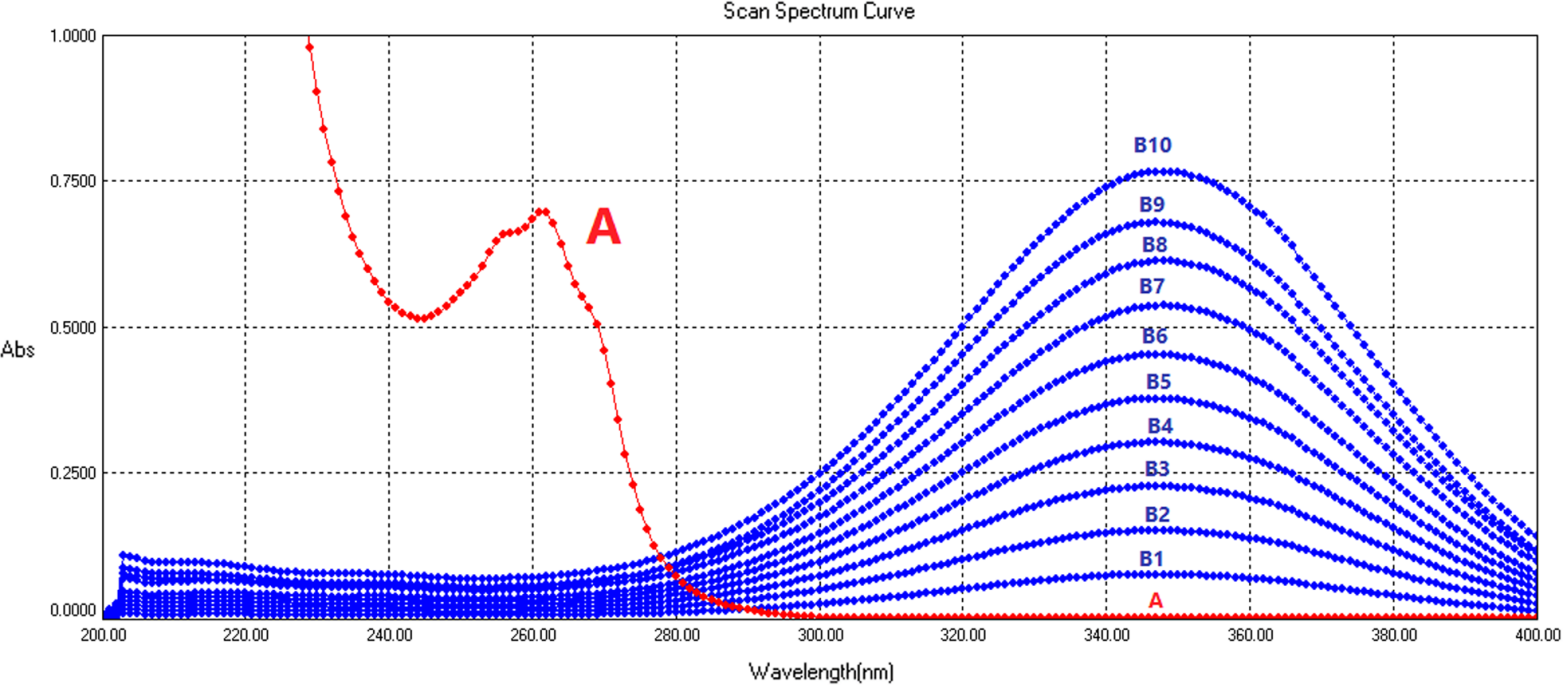
The zero-order UV absorption spectrogram in methanol shows (A) nicotinamide (30 µg/mL), and its 262 nm peak is overlapped by (B1–B10) tretinoin standard series (B1 = 0.5 µg/mL, B2 = 1.0 µg/mL, B3 = 1.5 µg/mL. B10 = 5.0 µg/mL) with 348 nm peaks

**Fig. 4:**
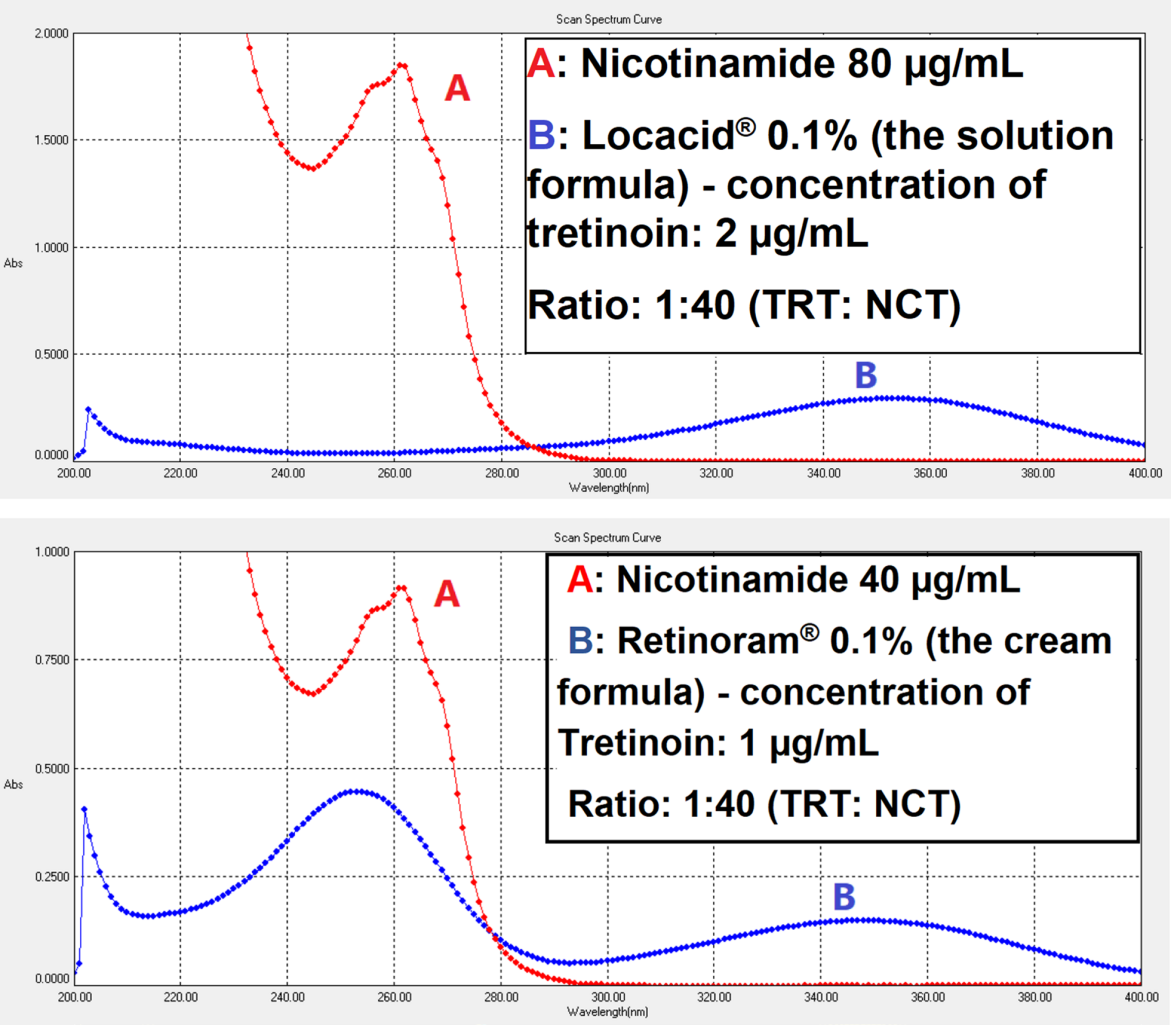
The zero-order UV absorption spectrograms show spectra of nicotinamide (A) and pharmaceutical preparations formulas of tretinoin (B) in methanol

UV spectrogram scan of pure NCT solution, pure TRT solution, and the binary mixture of both; using methanol as solvent appear in Fig. 2. We may notice two problems. The first one is that although the TRT absorption area has a wide region with no absorption of NCT or any excipients of TRT pharmaceutical formulas, the NCT absorption area is overlapped by TRT as mentioned in the last paragraph of the introduction and shown in Fig. 3 where a spectrogram of TRT linearity series are also shown in this figure. NCT is also overlapped by the absorption of the pharmaceutical formulas of TRT especially the cream as shown in Fig. 4. The second one is that NCT with a concentration of 80 µg/mL has absorption around the limit of linearity range at the zero-order (D_0_) 262 nm peak because of the high absorptivity of NCT at this wavelength; thus, first and second orders derivatives (D_1_ and D_2_) are needed and produced. Zero-crossing points of TRT and pharmaceutical preparations have been found in D_1_ and D_2_ for the determination of NCT at those points. Thus, we can solve the overlapping problem and determine NCT in the mixtures with concentrations that may exceed 120 µg/mL, yet they stay under the upper limit of the linearity range. While TRT concentration can be determined in zero-order at 348 nm as had been analyzed in many other studies [10, 11, 28–30].

**Fig. 5:**
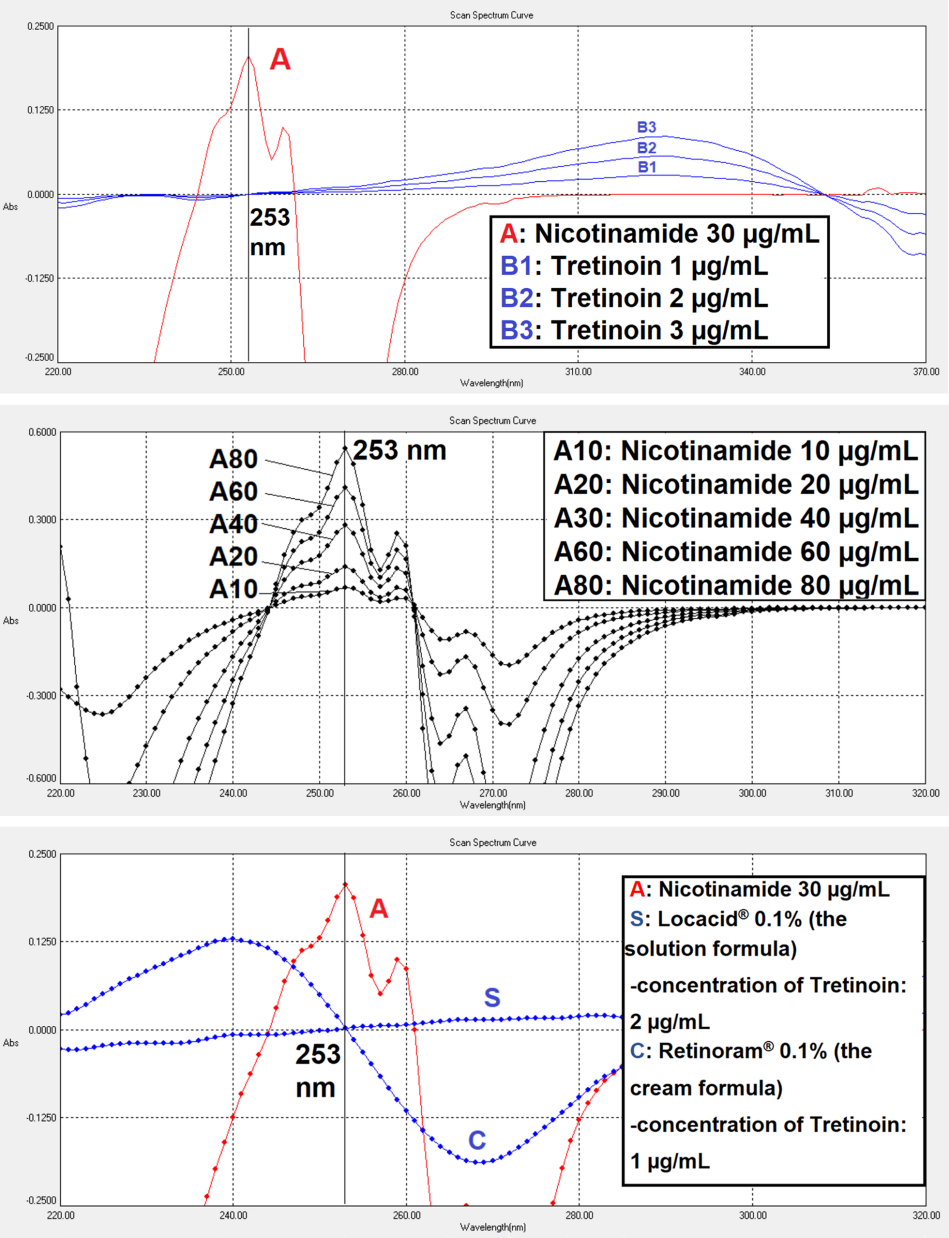
The first-order derivative spectrograms show nicotinamide spectrum 30 µg/mL (A), nicotinamide series (A10–A80), tretinoin standard series (B1–B3), solution formula (S), and cream formula (C)

For the first-order derivative (amplified 10 times [coefficient × 10]), wavelength 253 nm is a zero-crossing point for TRT and its pharmaceutical preparations spectra. While NCT has a sharp peak at this wavelength as shown in Fig. 5. A low concentration of 30 µg/mL of NCT was used for the demonstration. For TRT, concentrations in the middle of its range 1.0–3.0 µg/mL were used for the demonstration.

**Fig. 6:**
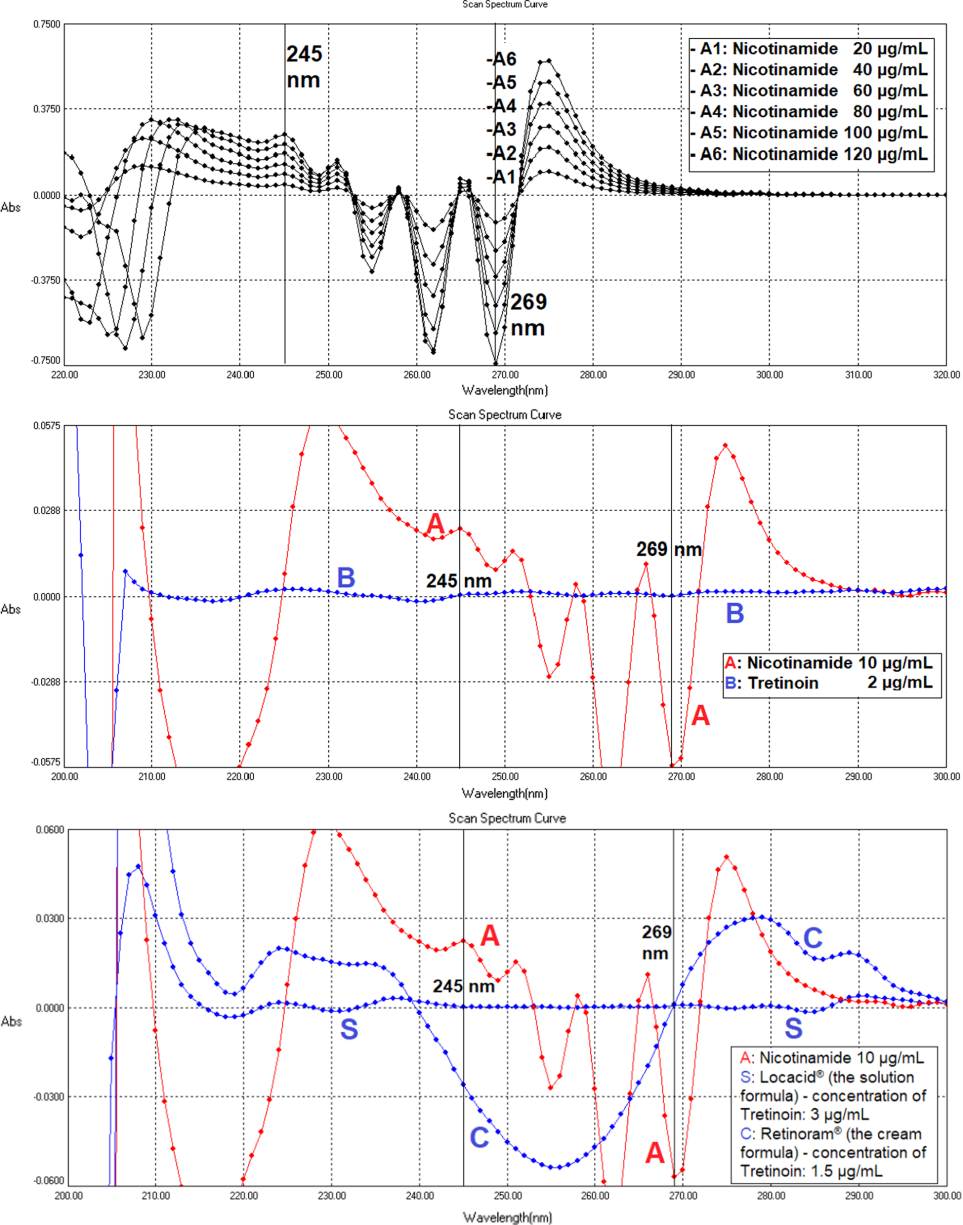
The second-order derivative spectrograms show nicotinamide spectrum 10 µg/mL (A), nicotinamide series (A1–A6), tretinoin 2 µg/mL (B), solution formula (S), and cream formula (C)

For the second-order (amplified 10 times [coefficient × 10]), more than two wavelengths were found to be zero-crossing points for TRT and its preparations spectra. Only two of them were selected (245 nm and 269 nm), where NCT has a sharp peak as shown in Fig. 6. However, 245 nm was not a zero-crossing point for the cream preparation due to its excipients; thus, 269 nm is the only wavelength for the cream formula.

### Method validation

#### Linearity

The spectra of NCT and TRT were taken and NCT linearity was studied at three wavelengths, which are D_1_ 253 nm, D_2_ 245 nm, and D_2_ 269 nm, while TRT linearity was studied at D_0_ 348 nm. D_1_ and D_2_ are amplified by 10 times for better resolution in the whole study. Additionally, correlation coefficient (r^2^), the limit of detection (LOD), and the limit of quantification (LOQ) were found. Results are shown in Table 1. We may notice from the results; accepted linearity ranges and correlation coefficients, and low LOD—LOQ for both NCT in the three wavelengths and TRT. Data were collected and processed according to ICH procedures, except for the stability and robustness studies [31].

**Table 1:** Analytical performance data for the proposed method

Component parameter	Nicotinamide			Tretinoin
Wavelength (nm), Dev order	253, D_1_	245, D_2_	269, D_2_	348, D_0_
Linearity-range (μg/mL)	10–80	20–120	10–120	0.5–5.0
Regression equation	y = 0.0068x + 0.0050	y = 0.0022x + 0.0037	y = 0.0061x—0.0028	y = 0.1556x + 0.0003
Correlation coefficient r^2^	0.9996	0.9998	0.9999	0.9999
LOD μg/mL	4.50	5.60	1.50	0.007
LOQ μg/mL	13.60	16.80	4.30	0.021

#### Stability

The stability was studied at room temperature (25 ℃), at low relative humidity, and in a dark flask for both NCT and TRT using methanol as solvent. However, NCT is already known for its good stability in both aqueous and alcoholic solutions. NCT 1 mg/mL in methanol can be purchased and transported worldwide by many suppliers. Our study shows that NCT is stable in methanol in storage at room temperature for more than 1 week [32].

The results of the TRT stability study are shown in Table 2. They show that TRT is stable in methanol in storage at room temperature for less than 3 days. This study was done for 3 concentrations of 0.75, 1.50, and 2.25 µg/mL each day.

**Table 2:** Data of the TRT Stability Study

TRT concentrations (µg/mL)	Percentage%			
	t_0_ = 0 h	t_1_ = 24 h	t_2_ = 48 h	t_3_ = 72 h
0.75	100.00	99.91	101.44	103.05
1.50	100.00	99.60	101.19	102.20
2.25	100.00	99.82	100.85	102.33
In the accepted range 98–102%		Yes	Yes	No

#### Accuracy

Accuracy study was achieved for TRT at D_0_ 348 nm and NCT at D_1_ 253 nm, D_2_ 245 nm, and D_2_ 269 nm. Three concentrations and three repetitions for each were analyzed at each of the previous wavelengths. Table 3 shows the mean, the relative standard deviation (RSD%), and the range of percentage values of the total 9 samples of NCT and TRT.

**Table 3:** Data of the Accuracy Study of NCT and TRT for the Proposed Method

NCT taken concentration (µg/mL)	NCT mean percentage ± RSD %^a^			TRT taken concentration (µg/mL)	TRT mean percentage ± RSD%^a^
Wavelength, Dev order	253 nm D_1_	245 nm D_2_	269 nm D_2_		348 nm D_0_
30	101.22 ± 0.72	99.10 ± 0.90	100.92 ± 0.68	0.75	100.49 ± 1.85
60	101.39 ± 0.63	101.48 ± 0.55	101.06 ± 0.64	1.50	100.40 ± 1.47
90	99.68 ± 0.57	101.30 ± 1.34	100.35 ± 0.07	2.25	99.78 ± 0.91
Mean percentage ± RSD%^b^	100.76 ± 0.98	100.63 ± 1.21	100.78 ± 0.57	Mean percentage ± RSD %	100.22 ± 1.67
Range%^b^	99.03–102.12	98.62–101.87	100.27–101.71	Range %	98.63–101.89

^a^Three repetitions of each concentration were analyzed with nine total repetitions

^b^From all 9 repetitions of each wavelength

#### Precision

The precision study was achieved for TRT at D_0_ 348 nm and NCT at D_1_ 253 nm, D_2_ 245 nm, and D_2_ 269 nm. Three concentrations with three repetitions for each were analyzed at each of the previous wavelengths for the intraday repeatability precision. The same number of samples were analyzed after 24 h and again after 48 h for the intermediate inter-day precision with a total of 27 samples. RSD% values for all cases are shown in Table 4.

**Table 4:** Data of the Precision Study of NCT and TRT for the Proposed Method

Precision	NCT RSD%			TRT RSD%
Category^a^	253 nm D_1_	245 nm D_2_	269 nm D_2_	348 nm D_0_
Intraday	0.73	1.02	0.92	0.76
Inter-day 24 h	1.49	0.62	0.97	1.07
Inter-day 48 h	1.89	0.98	1.19	1.51
total repetitions	1.41	0.90	1.00	1.11

^a^For each category: there are three concentrations and three repetitions for each concentration with a total of 27 repetitions

#### Specificity

Specifity studies were achieved by using the mixture of NCT and TRT with several ratios for the same wavelengths as previous Sections “Accuracy” and “Precision” results are shown in Table 5. The method has good recovery values for a wide range of the ratio of the binary mixture contents compared to the most used ratio of 1:40 (TRT: NCT).

**Table 5:** Data of the Specifity Study of NCT and TRT for the Proposed Method

Ratio (TRT: NCT)	Tretinoin concentration (µg/mL)	Nicotinamide (80 µg/mL) recovery%		
		D_1_ 253 nm	D_2_ 245 nm	D_2_ 269 nm
1:40	2.0	100.00	100.00	100.00
1:20	4.0	99.42	100.29	100.70
1:27	3.0	99.77	100.47	99.11
1:80	1.0	99.39	100.63	100.11
1:160	0.5	100.24	100.58	99.85
Range %		99.39–100.24	100.29–100.63	99.11–100.70

#### Robustness

A robustness study was achieved for TRT and NCT in only D_2_ 245 nm for three different instrumental parameters changes and three repetitions for a minor change in each parameter independently. First, the scan speed was changed from medium to fast. Second, the scan range was changed from 200–400 nm to 190–350 nm. Third, the bandwidth of the light beam was changed from 0.2 nm wide to 0.1 nm wide. The developed method appeared to be robust for TRT with the three tests but only robust with two of them for NCT. Recovery varied when the bandwidth of the spectrometer was changed. That was probably due to dependence on its strict method of zero-crossing point. NCT had recovery ranges of: 98.13–101.91% for scan speed parameter, 100.26–101.91% for scan range parameter, and 102.56–116.10% for bandwidth parameter. TRT had recovery ranges of: 100.16–101.42% for scan speed parameter, 99.67–100.84% for scan range parameter, and 98.33–99.35% for bandwidth parameter.

### Application of the proposed method for pharmaceutical preparations

The pharmaceutical preparations were analyzed for their content of TRT and the content of the added NCT from standard solutions. In this study standard additions method has been used for both NCT and TRT with three repetitions. All amounts and concentrations that were prepared for this test are mentioned in Section “Preparation of synthetic mixture from pharmaceutical formulas”.

#### Dermal solution preparation

The recovery% and RSD% of both TRT and NCT for each selected wavelength are shown in Table 6. As shown, the recovery values are within the accepted range in ICH guidelines.

**Table 6:** Data of the analysis of nicotinamide and tretinoin in the synthetic pharmaceutical preparation with the Proposed Method

Ingredient/labeled concentration (w/w%)	Nicotinamide (4%)			Tretinoin (0.1%)
Wavelength (nm)/deriv. order	253/D_1_	245/D_2_	269/D_2_	348/D_0_
Recovery ± RSD %^a^	101.00 ± 0.90	100.40 ± 1.22	99.30 ± 0.88	93.70 ± 1.39
Recovery ± RSD %^b^	101.70 ± 1.42	–	101.16 ± 1.62	98.37 ± 0.90

^a^In solution preparation

^b^In cream preparation

TRT in pharmaceutical preparations has an accepted assay range of 90–120% in several pharmacopeias like the British Pharmacopeia (BP) and the United States Pharmacopeia (USP) because the concentration of TRT in the preparations is 0.1% or less [33, 34].

#### Dermal cream preparation

The recovery% and RSD% of both TRT and NCT for each selected wavelengths are shown in Table 6. The studied cream has no zero-crossing point around 245 nm D_2_ as we mentioned before in Section “First and second-order derivative spectrophotometric optimal wavelength” thus NCT couldn't be studied at that point.

## Conclusion

It could be concluded from the results obtained in the present paper that the developed method for simultaneous determination of NCT and TRT in binary mixtures is simple, accurate, robust, precise, and rapid. This method can be used for routine quality control tests to directly determine NCT and TRT individually or simultaneously in a binary combination of either solution or cream formula with their excipients in the mixture without any prior separation. Moreover, it's recommended to analyze TRT within 48 h after being dissolved in methanol because TRT is stable within this period according to the stability test.

## Data Availability

Data and materials are included in this manuscript.

## Abbreviations

BP: British pharmacopeia
D_0_: Zero-order spectrum
D_1_: First-order derivative
D_2_: Second order derivative
HPLC: High-performance liquid chromatography
LOD: Limit of detection
LOQ: Limit of quantification
NCT: Nicotinamide
PP: Pellagra preventing substance
r^2^: Correlation coefficient
RARs: Retinoic acid receptors
RSD%: Relative standard deviation
TRT: Tretinoin
USP: United States pharmacopeia
UV: Ultra-violet
λ_max_: Wavelength with maximum absorbance
